# The last *Palaeoproteus* (Urodela: Batrachosauroididae) of Europe

**DOI:** 10.1038/s41598-020-59255-1

**Published:** 2020-02-17

**Authors:** Davit Vasilyan, Vadym Yanenko

**Affiliations:** 1 JURASSICA Museum, Route de Fontenais 21, 2900 Porrentruy, Switzerland; 2 Department of Geosciences, University of Fribourg, Chemin du musée 6, 1700 Fribourg, Switzerland; 3 Department of Paleontology, National Museum of Natural History, National Academy of Sciences of Ukraine, Bogdan Khmelnitsky st. 15, 01030 Kyiv, Ukraine

**Keywords:** Evolution, Evolution, Herpetology, Herpetology, Palaeontology

## Abstract

The Batrachosauroididae are an enigmatic group of salamanders known from the Cretaceous and Tertiary of North America and Europe. In Europe, the family is known only by two species of the genus *Palaeoproteus*. The genus has limited distribution in Western and Central Europe. In the present paper, we describe a new species, *Palaeoproteus miocenicus*, from the early late Miocene (11–9 Ma) of Austria and Ukraine, representing the youngest record of the family Batrachosauroididae from the Neogene of Europe. The new species differs from the Paleogene representatives of the genus by 12 characters, including large body size, the long anterior extension of the Meckelian groove and the size and shape of the odontoid process on the dentary. The µCT scanning of bones of the new species revealed novel features (e.g. anterior extension of Meckelian groove, interconnected network of canals and small cavities in atlas) observable only in this species. *P. miocenicus* inhabited aquatic environments, which existed under wet climatic conditions with mean annual precipitation higher than 900 mm. The new species expands the temporal range of the genus by at least 30 million years and enlarges the palaeogeographic distribution of the genus into Eastern Europe.

## Introduction

The present-day European salamander fauna is represented by the Salamandridae, Plethodontidae, Proteidae and Hynobiidae families^1,2^. Besides these families, the Cenozoic record of European salamanders includes also the extant family of Cryptobranchidae^3^, as well as several enigmatic salamanders such as Batrachosauroididae^4^, an ambistomoid^5^, *Seminobatrachus*^6^, *Bergmannia*^7^, *Geyeriella*^8^ and *Wolterstorfiella*^8^. All these groups are known nearly exclusively from the Paleogene Period. Phylogenetic analysis of *Seminobatrachus* gives equivocal results, but always nests it within the *Salamandra* + Ambystomatidae + *Dicamptodon* + *Rhyacotriton* clade^6^. The genera *Bergmannia*, *Geyeriella* and *Wolterstorfiella* have been assigned to the subfamily Dicamptodontinae^4^, although these relationships have not been tested with a phylogenetic analysis. Though the phylogenetic relationships of many groups are not well resolved, affinities between North American and European Cenozoic salamander groups are widely recognized^4,9^.

The first fossil record of the family Batrachosauroididae is known since the Cretaceous of both Northern America and Europe. In North America the family is highly diverse, containing five genera (*Batrachosauroides*, *Opisthotriton*, *Parrisia*, *Peratosauroides*, *Prodesmodon*) ranging from Late Cretaceous (Cenomanian) to Pliocene^10^. In Europe, the Batrachosauroididae include only one genus, *Palaeoproteus*, known by fossils from Paleocene and Eocene localities in Western (France) and Central (Germany) Europe^4,11]^. Currently known occurrences suggest the family Batrachosauroididae has been extinct in Europe since the late Eocene.

**Figure 1:**
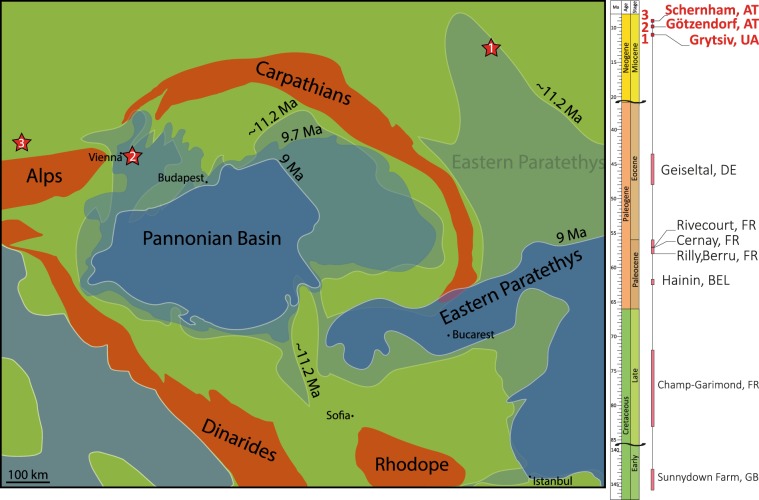
Palaeogeographic map during late Miocene depicting with the changing shorelines of the Paratethyan Sea and Pannonian Lake. Maps redrawn from Popov *et al*.^41^ and Uhlin & Sztanó^42^. The numbers in the red stars correspond to the localities: 1 Grytsiv, 2 Götzendorf and 3 Schernham. Stratigraphic distribution of the batrachosauroid fossil record from Cretaceous to Neogene in Europe. Details on the localities see Table 1.

In the present paper, we document the fossil record of the genus *Palaeoproteus* from the Neogene and describe a new species based on disarticulated bones from three late Miocene age localities in Central (Götzendorf and Schernham, Austria) and Eastern (Grytsiv, Ukraine) Europe (Fig. 1).

## Geological Settings

### Grytsiv, western Ukraine

The locality Grytsiv represents a karstic filling deposited at the coastal line of the Eastern Paratethys Sea during the Bessarabian, late Tortonian. The fossil layer is characterized by greenish clays located in the karstified cavities of the lower Bessarabian limestone. The karstic fillings are covered by the middle Bessarabian marine clays (for details see Vasilyan *et al*.^12^, Fig. 1B).

The stratigraphic position of the fossil layer, its high content of vertebrate remains and the grain size likely suggest deposition of these clays in open holes of the underlying limestone under terrestrial conditions. The small mammalian assemblage, found in the Grytsiv site, correlates to Lower Vallesian^12,13^. The fossiliferous clays have reversed polarity^14^ that together with other biostratigraphic markers can be correlated to the chron C5r (11.146–11.056 Ma)^12,15^.

### Götzendorf, Austria

The locality Götzendorf is situated in the southern part of the Vienna Basin in Lower Austria. The deposits are part of the lower sequence of the Upper Pannonian Neufeld Formation, Central Paratethys. The fossil material comes from cross-bedded fine sand enriched by carbonate concretions, mollusc shells, reworked meadow loam, lignite and rubble. The depositional environment for the fossiliferous bed was interpreted as floodplains and wetlands^16^. The age of the fossil beds has been dated by assemblages of its molluscs of the *Mytilopsis neumayri* Zone and small mammals (early Vallesian) at 9.7–9.9 Ma^17^.

### Schernham, Austria

The locality Schernham is located in the Northern Alpine Foreland Basin, Upper Austria and is confined to sediments of the Upper Freshwater Molasse, Western Paratethys. The fossiliferous horizon of Schernham is represented by greyish-yellow fine- to coarse-grained sands enriched with organic material (fluvial reworked sediments). The depositional environment has been interpreted as a system of high-energy braided rivers that submerged the river-bank, floodplain and adjacent forest area during flooding periods^18^. The age of the Schernham beds has been dated at 8.9–9.1 Ma using the small mammal assemblage indicative of the second part of the late Vallesian^17^.

### Systematic palaeontology

Lissamphibia Haeckel^19^

Caudata Scopoli^20^

Urodela Dumériel^21^

Batrachosauroididae Auffenberg^22^

*Palaeoproteus* Herre^23^

**Figure 2:**
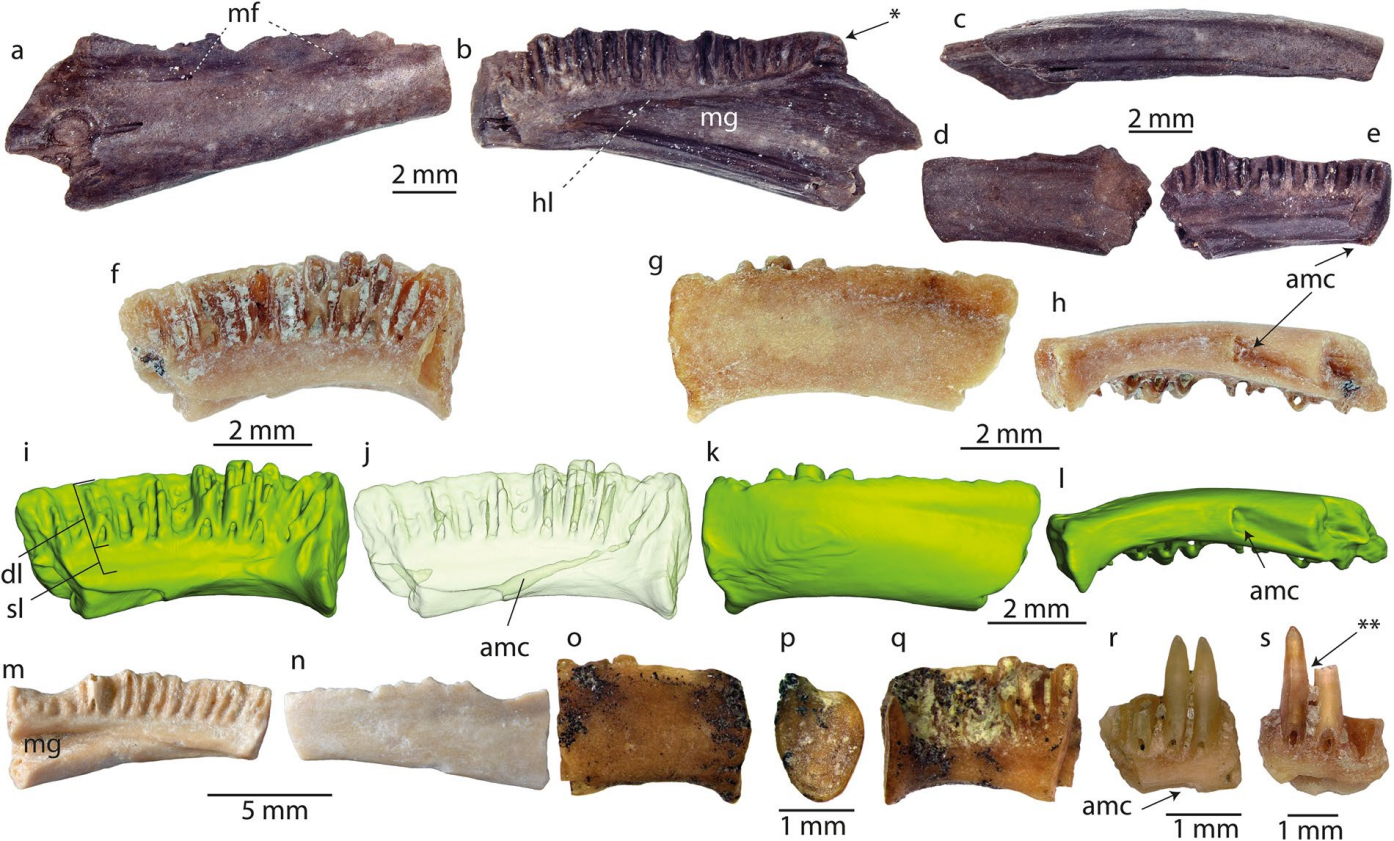
Dentary remains of *Palaeoproteus miocenicus* sp. nov. from the Grytsiv (**a–e**) and Schernham (**f–s**) localities. (**a–h,m–s**) photographs and (**i–l**) 3D models of the bones. (**a–c**) right dentary, loc. Grytsiv (NHMHU-P 22–2705); (**d,e**) left dentary, loc. Grytsiv (NHMHU-P 22–2707), (**f–aa**) the holotype of *P. miocenicus*, left dentary, loc. Schernham (NHMU 2018/0290/0001). (**m**,**n**) left dentary (NHMW 2018/0290/0006). (**o–q**) Right dentary (NHMW 2018/0290/0009), (**r**) (NHMW 2018/0290/0007) and (**s**) (NHMW 2018/0290/0008) dentary fragments with dentition, loc. Schernham. (**a,d,g,k,n,o**) labial, (**b**,**e**,**f**,**i**,**j**,**m**,**q–s**) lingual, (**c,h,l**) ventral and **m** symphyseal views. **j** 3D model with 80% surface transparency. Abbreviations: amc, anterior opening/extension of the Meckelian canal; arc, anterior cotyle; dl, dental lamina; lh, lamina horizontalis; mc, Meckelian groove; mf, mental foramen; sl, subdental lamina. One asterisk (*) points the bulb of the coronoid process, two asterisks (**) point the transition between the tooth crown and tooth pedicle.

**Figure 3:**
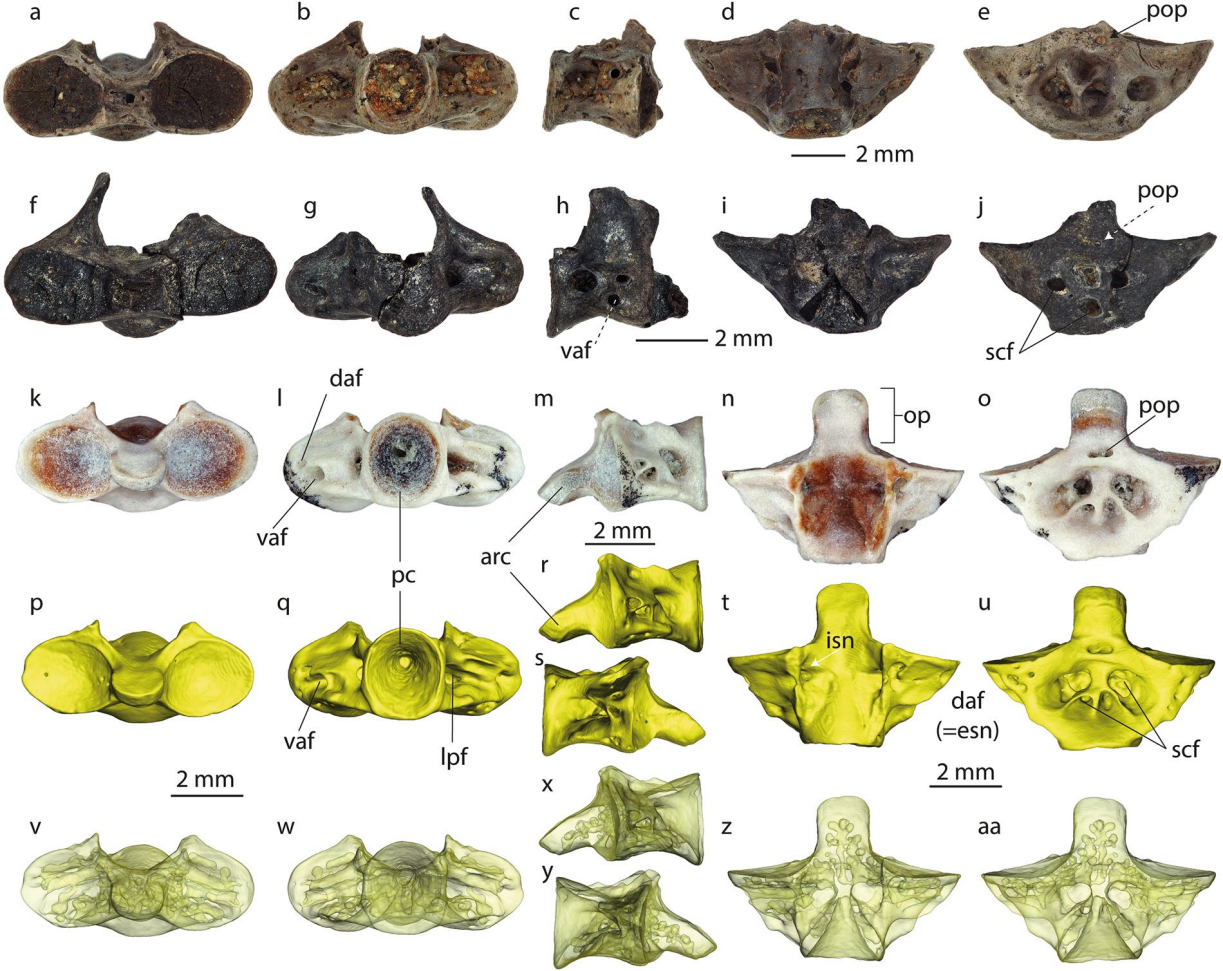
Atlantal remains of *Palaeoproteus miocenicus* sp. nov. from the Götzendorf (**a–j**) and Schernham (**k**–**aa**) localities. (**a–o)** photographs and p-aa 3D models of the bones. (**a–e**) NHMW 2000z0196/0004, (**f–j**) NHMW 2000z0196/0003. (**k**-**aa**) NHMW 2018/0290/0002. **v–aa** with 80% surface transparency. Abbreviations: daf, dorsoanterior foramen; esn, external opening of the spinal nerve; isn, internal opening of the spinal nerve; lpf, large posterior foramina; op, odontoid process; pc, posterior cotyle; pop, posterior process; scf, subcentral foramina; vaf, ventroanterior foramen.

**Figure 4:**
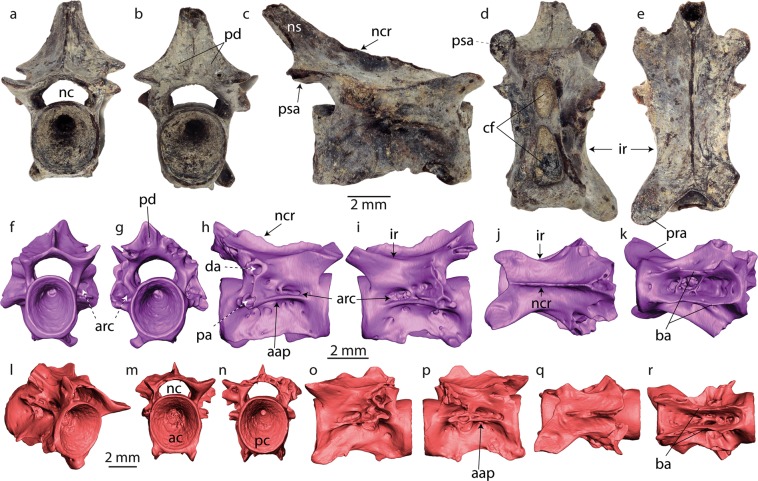
Trunk vertebrae of *Palaeoproteus miocenicus* sp. nov. from the Grytsiv (**a–e**) and Schernham (**f–r**) localities. (**a–e**) photographs and (**f–r**) 3D models of the bones. (**a–e**) NMNHU-P 22–2708, (**f–k**) NHMW 2018/0290/0003, (**l–r**) NHMW 2018/0290/0004. (**a,f,m**) anterior, **(b,g,n**) posterior, (**c,h,i,o,p**) lateral, (**d,k,r**) ventral, (**e,j,q**) dorsal and **l** anterolateral views. Abbreviations: ac, anterior cotyle; aap, anterior alar process; arc, arterial canal; ba, basapophysis; cf, central foramina; da, diapophysis; ir, interzygapophyseal ridge; nc, neural canal; ncr, neural crest; ns, neural spine; pa, parapophysis; pc, posterior cotyle; pd, posterior depression; pra, prezygapophysis; psa, postzygapophysis.

**Figure 5:**
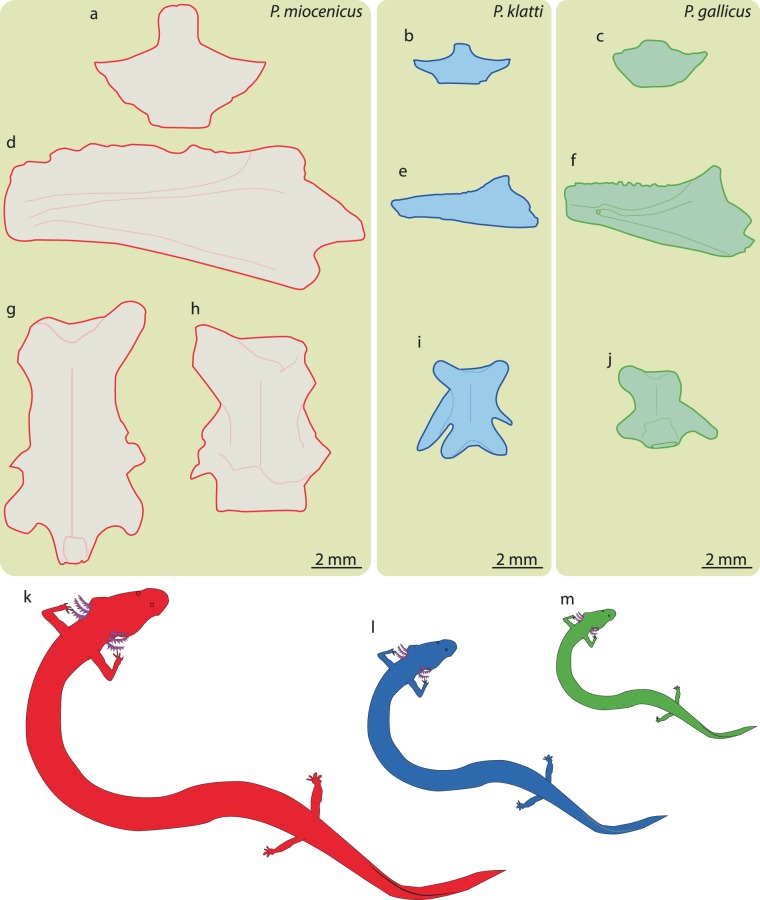
Comparison of skeletal elements (**a–c** atlas, **d–f** dentary and **g–j** trunk vertebra) and relative body sizes (**k–m**) of *Palaeoproteus miocenicus* (**a,d,g,h,k**), *P. klatti* (**b,e,i,l**) and *P. gallicus* (**c,f,j,m**). Bone outlines have been redrawn for *P. miocenicus* according to the present study, *P. klatti* according to Herre^23^, *P. gallicus* according to Estes *et al*.^11^.

*Palaeoproteus miocenicus* sp. nov.

(Figures 2–5a,d,g,k)

2002 Caudata indet. Miklas^24^, p. 190.

#### Holotype

Incomplete left dentary NHMW 2018/0290/0001, Fig. 2f–aa, Naturhistorisches Museum Wien, Austria.

#### Paratypes

Incomplete right dentary NHMW 2018/0290/0006, jaw fragment with teeth NHMW 2018/0290/0007, one atlas NHMW 2018/0290/0002; one trunk vertebra NHMW 2018/0290/0003.

#### Type locality

Schernham, gravel pit Schernham bei Haag am Hausruch, Upper Austria. 48.1500 N, 15.5000 E.

#### Type horizon

Greyish-yellow fine- to coarse-grained sands enriched on organic material^18^.

#### Stratigraphy and age

Upper Freshwater Molasse, Upper Vallesian, 8.9–9.1 Ma, Tortonian Stage, late Miocene^17^.

#### Referred material

Loc. Grytsiv: three incomplete dentaries NMNHU-P 22–2705; NMNHU-P 22–2706; NMNHU-P 22–2707; one trunk vertebra NMNHU-P 22–2708. Loc. Götzendorf: three incomplete atlases NHMW 2000z0196/0002 - /0004. Loc. Schernham: 18 incomplete dentaries NHMW 2018/0290/0008 - /0025; two trunk vertebrae NHMW 2018/0290/0004 – /0005.

#### Diagnosis

*Palaeoproteus miocenicus* differs from other *Palaeoproteus* spp. in having: (at least 1.4 times) larger body size; one or two small-sized, elongate mental foramina located labially along the posterior half of dentary; weakly-developed subdental shelf on dentary; Meckelian groove with anteroventral extension, terminating by ventrally located foramen; dentary bearing weakly-developed coronoid process; dentary symphysis with medial semi-oval extension; external surface of atlas pierced by many foramina of different sizes; postodontoid foramen present; anterior cotyles of atlas oval in outline, slightly anterodorsally compressed and weakly concave; strongly pronounced, lip-shaped odontoid process on atlas. The new species further differs from *P. klatti* in having a higher/longer neural spine (condition unknown in *P. gallicus*).

#### Etymology

The specific name derives from the Miocene Epoch, during which the salamander species existed.

#### Dentaries

The preserved dentaries from Grytsiv and Schernham are represented by individuals of different sizes **(**Fig. 2**)**. The height of the dentaries measured at its most narrow postsymphyseal portion varies from 1.5 to 3 mm. The bone is elongate and low. Its posteroventral portion extends ventrally. The holotype (NHMW 2018/0290/0001) from Schernham is the anterior part of a dentary, with eleven tooth positions and an incompletely-preserved symphysis (Fig. 2i–l). The fragmentary symphysis has an oval outline and possesses a narrow transversely projecting (lateroventrally to mediodorsally) depression separating the medial extension of the symphysis from the dentary body. In lingual view, the pars dentalis (sensu Vasilyan *et al*.^12^) is thrice as high as the corpus dentalis. The pars dentalis is composed of the high dental lamina and reduced subdental lamina. The dental lamina possesses eleven tooth pedicles. They have thick walls and are strongly anteroposteriorly compressed. The bases of the pedicles are pierced by foramina. The lamina horizonalis is moderately pronounced. The anterior extension of the Meckelian groove is observable on the preserved dentary fragment. Anteriorly the Meckelian groove narrows and pierces the bone at the level of the tenth-eleventh tooth positions (Fig. 2). The anterior portion of the Meckelian groove is placed at the lingual and linguoventral surfaces of the dentary. It has the form of an anteroventrally oriented shallow depression. The depression disappears on the ventral surface of the bone by a foramen at the level of the seventh tooth position. This foramen is the opening of an anterodorsally running canal. The latter has an anterior opening located in the centre of the pedicle of the second tooth. In labial view, the dentary has a smooth surface, no foramen is visible. Only a shallow groove is visible at the dorsal portion of the bone. It runs subparallel to the dorsal margin of the bone and enlarges posteriorly.

The only completely preserved symphysis (NHMW 2019/0290/0009) is oval in outline. The symphyseal face is semicircular in shape (Fig. 2p). The anteroventral foramen of the Meckelian groove is located at the level of the fifth-seventh tooth positions (Fig. 2i,q).

All dentary specimens lack the posterior part of the bone. In lingual view, the pars dentalis lies above the subdental shelf sensu Vasilyan *et al*.^12^. It consists of strongly widened dental and narrow subdental laminae. The dental lamina possesses traces of pleurodont teeth. They are arranged in one row. The pedicles in cross-section have thick walls and are ellipsoid in outline. The pars dentalis as well as the dental lamina increase in height anteriorly. The lamina horizontalis is high anteriorly but reduces in height posteriorly. Both pars dentalis and lamina horizontalis terminate posteriorly in a moderately developed bulb (Fig. 2b, indicated by an asterisk). The Meckelian groove is expanded and has a concave surface (C-shaped in cross-section). Anteriorly, it narrows significantly and continues anteroventrally as a shallow but rather broad tract. The lingual surface of the corpus dentalis does not possess any foramina or pits. The posteroventral part of the bone extends strongly ventrally. In labial view, there is a shallow longitudinal groove along the dorsal edge of the bone. Several small mental foramina are visible in the posterior part of the bone (NMNHU-P 22–2705, Fig. 2a). The coronoid process is reduced. The posteroventral surface of the dentary possesses an anteriorly reducing shallow and narrow ventral groove, which can be pierced by small foramina. Two dentary fragments with pleurodont teeth (Fig. 2r,s) are preserved. The teeth are pedicellated with very smooth transition from the pedicle to the tooth crown (Fig. 2s). The tooth crown is pointy, long and has smooth surface without any structures. Their tips are curved slightly lingually and show slight linguolabial (NHMW 2018/0290/0008; Fig. 2s) or nearly (NHMW 2018/0290/0007; Fig. 2r) no compression.

#### Atlas

All four atlases lack neural arches; only the vertebral centra are present (Fig. 3**)**. In dorsal and ventral views, the centrum of the atlas is triangular in shape. The sizes (width) of the studied vertebrae vary between 6.5 mm (loc. Schernham, NHMW 2018/0290/0002) to 9.5 mm (loc. Götzendorf, NHMW 2000z0196/0004) (Fig. 3). The internal structure of the centrum consists of an interconnected network of canals and small cavities. All observable foramina at the surface of the atlases, excepting the postodontoid foramen, represent the external openings of this network. The odontoid process is pierced by a longitudinal canal with cavities branching out from it. In ventral view, the atlas centrum possesses several large and small subcentral foramina, all are located in a shallow broad depression. The lateroventral margins of the atlas are pierced by a row of smaller foramina. In all studied atlases, at the border of the odontoid process and the centrum, a postodontoid foramen of variable sizes is observable. The cavity opening from this foramen into the atlas does not connect with the main network of the centrum, at least in the Schernham vertebra (NHMW 2018/0290/0002). In anterior view, two distinct anterior cotyles are visible. They are oval in outline and weakly concave. The surfaces of the anterior cotyles are also pierced by small foramina. The articulation surfaces of both anterior cotyles are connected through the articular condyle of the odontoid process. In lateral view, a distinct anteroventrally oriented odontoid process is visible. Its dorsal surface is flat, but the ventral surface is convex. The articulation surface of the articular condyle is located laterally and ventrally at its most anterior tip. The lateral walls of the centrum are pierced by one large posterior and two medium-sized, closely located anterior foramina. The dorsally located medium-sized foramina open medially at the anterolateral corners of the neural canal. Most probably, these foramina represent the external and internal openings for the spinal nerve respectively. The neural arch is broken off. Only its bases are present. Laterally at the midpoint of the bases of the neural arch, distinct depressions are visible. The dorsal surfaces of the centrum, behind the anterior cotyles, possess lateromedially oriented slightly concave surfaces, which connect with the distinct depressions. In posterior view, the posterior cotyle is subcircular. The inner walls of the cotyles are lined with cartilage. The notochordal pit is present. The large posterior foramina are located laterally at its midline.

#### Trunk vertebrae

In later views, the trunk vertebrae are rectangular in shape **(**Fig. 4**)**. The vertebral centrum is amphicoelous. The sizes of the studied centra vary from 5.3 to 7 mm in length (Table S1) and from 2.4 to 3.2 mm in width. The estimated total body and snout-vent lengths (using the centrum length of trunk vertebra) range from 182 to 411 mm and from 133 to 300 mm, respectively. In anterior and posterior views, the cotyles are subcircular and compressed slightly laterally. The centrum is dumbbell-shaped in lateral view. The lateral and ventral surfaces of the centrum are pierced by many foramina of different sizes. Two (NMNHU-P 22–2708) or three (NHMW 2018/0290/0004) large central foramina are present along the longitudinal axis of the centrum between the paired, anterior basapophyses. Further small- or medium-sized foramina can be found around the large ones (subcentral foramina) and on the lateral surfaces of the centrum. The basapophyses are represented as thin walls, which reach the highest height at the centrum centre. The basapophyses are oriented either subparallel along their entire length (NHMW 2018/0290/0003, Fig. 4k) or posteriorly they run subparallel and anteriorly are curved slightly laterally (NHMW 2018/0290/0004 [Fig. 4r], NMNHU-P 22–2708 [Fig. 4d]). The basapophyses of the vertebra with the first morphology show posterior extensions, whereas the basapophyses of the second morphology show anterior extensions. The anterior extensions can project below the anterior border of the anterior cotyle.

The transverse process is preserved only on NHMW 2018/020/0003 by its proximal portion (Fig. 4h). It shows distinct posterolaterally oriented dia- and parapophysis that are connected by a thin ridge. The parapophysis connects anteriorly with the centrum by the horizontal projecting anterior alar process that forms a flange. Above the flange and at the anterior and posterior bases of the transverse process, the openings of the arterial canal of irregular shapes are observable.

The neural canal is low and compressed dorsoventrally. The neural arch is flattened and possesses a low neural crest. It runs from the anterior margin of the neural arch and terminates at the tip of the neural spine. The prezygapophyses are oriented laterodorsally, whereas the postzygapophyses are horizontal. The articulation facies of the prezygapophyses are elongate, whereas those of the postzygapophyses are round. The prezygapophysis is connected with the diapophysis by a weak interzygapophyseal ridge. The neural spine is high (Fig. 4f). Its posterior surface possesses two posterior shallow depressions separated by a low ridge running along the sagittal axis of the vertebra.

#### Body size reconstruction

The body size of the new species, reconstructed based on the length of the vertebral centrum (see Supplementary Method D1 and Table S1), shows total length ranging from 17.3 to 39 cm, whereas the snout-vent length – from 12.6 to 28.4 cm (Table S1).

### Taxonomic identification

The studied vertebrae can be assigned to the family Batrachosauroididae by the following combination of characters: (1) anterior cotyles of the atlas large and weakly concave; (2) both anterior and posterior cotyles of the atlas subcircular in outline; (3) odontoid process of the atlas is variously present, but typically reduced to a shelf; (4) trunk vertebrae amphicoelous with subcircular cotyles; (5) spinal foramina absent in trunk vertebrae; (6) vertebra centrum with basapophyses; (7) neural spine projecting posterodorsally^4,10^. Though no batrachosauroidid characteristic features have been mentioned in the literature for the identification of the jaw remains, few characters can be listed, which commonly are observable on dentary material, e.g. see Estes and Hecht^4^, Estes^11^, referable to the family: (8) dentary elongate, with posteroventrally projecting ventral portion of the bone; (9) presymphyseal portion of the dentary convex and smooth; and (10) dental pedicles ellipsoid in outline with thick walls, a foramen visible at the lingual base of the pedicles. Though the described finds have been found as disarticulated bones, we consider them to belong to one form; since they have been found from the same fossiliferous horizons; they show similar preservation; represent individuals with similar size ranges and all having features characteristic for the genus *Palaeoproteus* and the family Batrachosauroididae.

Among the batrachosauroidid salamanders the fossil remains resemble the morphology of the genus *Palaeoproteus* in having a combination of the following characters: (1) amphicoelous trunk vertebrae with well-developed paired ventral basapophyses that unite posteriorly and diverge anteriorly^4^; (2) anterior cotyles of the atlas subcircular and (slightly) dorsoventrally compressed; (3) dentary with a distinct coronoid process. The third character can be stated only for three genera *Batrachosauroidides, Prodesmodon* and *Opisthotriton*, because for *Peratosauroides* the dentaries are not known. Most probably, further characters of the trunk vertebrae such as (4) a flattened neural arch and (5) a well-pronounced anterior alar process, developing a flattened ventral lamina between the anterior cotyle and parapophysis, are also unique characters for the genus *Palaeoproteus*. However, due to incomplete preservation of other fossils of batrachosauroidid genera and insufficient literature data, this cannot be stated.

The studied skeletal remains show clearly different morphology from the known fossil species of the genus *Palaeoproteus* in having:

1. *larger body size*. The snout-vent length in *P. klatti* ranges 86 to 198 ± 7 mm, whereas the reconstructed snout-vent length in *P. miocenicus* varies between 126 ± 5 to 284 ± 4 mm and in *P. gallicus* equals to 118 ± 4 mm. The width of the atlases of the new species ranges 6.5–9.5 mm, whereas in *P. gallicus* 3.7–4.5 mm and in *P. klatti* 3.1–5 mm; the length of the centra of the trunk vertebra ranges in *P. miocenicus* 6.4–7.8 mm, whereas in *P. gallicus* it is 3 mm and in *P. klatti* it is 3–3.5 mm (Fig. 5).
2. *One or two small-sized, elongate mental foramina located labially at the posterior half of the dentary. P. klatti* has no (personal observation of DV) or only one foramen^4^ at the labial surface of the dentary. In *P. gallicus* one large foramen is observable labially at the midpoint of the dentary.
3. *Weakly-pronounced subdental shelf on dentary. P. klatti* does not have a subdental shelf^23^, whereas it is well-pronounced and pierced with a foramen in *P. gallicus*^11^.
4. *Meckelian groove with anteroventral extension, terminating by ventrally located foramen*. In both *Palaeoproteus* species, the Meckelian groove terminates anteriorly into a lingually located foramen^11,23^.
5. *Dentary with weakly-developed coronoid process*. The coronoid process is large and tear-drop shaped in *P. gallicus*^11^. In *P. klatti* this process is less pronounced^23^ than in *P. gallicus* and shows an intermediate state between *P. gallicus* and the new species.
6. *Symphysis with medial semioval extension. P. klatti* lacks any symphyseal extension^23^. *P. gallicus*^11^ shows an elongate oval and relatively little expanded symphysis.
7. *External surface of the atlas is pierced by foramina of different sizes*. In Herre^23^ and Estes *et al*.^11^ figured atlases of *P. klatti* and *P. gallicus* possesses only few small foramina.
8. *Postodontoid foramen is present*. No foramen is observable on the dorsal surface of the atlas at the base of the odontoid process in *P. klatti*^23^ and *P. gallicus*^11^.
9. *Weakly concave anterior cotyles of the atlas*. Strongly concave anterior cotyles can be found in the atlases of both *P. klatti*^23^ and *P. gallicus*^11^ species.
10. *Anterior cotyles of the atlas are oval in outline and slightly anterodorsally compressed*. In *P. klatti*^4,23^ and *P. gallicus*^11^, these cotyles are subcircular in outline.
11. *Strongly pronounced, lip-shaped odontoid process of the atlas*. The odontoid process is broken off or small in *P. gallicus*^11^, whereas, it is small and ridge-like in *P. gallicus*^23^. In *P. miocenicus*, the odontoid process is very large in comparison to all known batrachosauroidids. We speculate that a large odontoid process could be an adaptation to larger body/head sizes, which will require more support.
12. *Higher and longer neural spine*. In *P. klatti* the neural spine is short and low^23^. In *P. gallicus* the neural spine is not preserved^11^.

Considering the listed twelve characters, the studied late Miocene *Palaeoproteus* remains from the Ukrainian (loc. Grytsiv) and Austrian (Götzendorf and Schernham) localities can be clearly distinguished from the Paleogene species *P. klatti* and *P. gallicus* and assigned to our new species *P. miocenicus*.

## Discussion and Conclusions

The new species *Palaeoproteus miocenicus* from three upper late Miocene localities in Europe is a unique member of the family Batrachosauroididae considering its taxonomy, stratigraphy and palaeobiogeography. The new species is significantly larger than the known *Palaeoproteus* species from the Paleogene. µCT imaging of some of the studied bones enabled documentation novel features of the new species which make *P. miocenicus* unique in comparison to other forms. So, *P. miocenicus* shows morphological differences from other *Palaeoproteus* spp., such as the anterior extension of the Meckelian groove and the size and shape of the odontoid process of the atlas. This points, most probably, that the new species represents a derived member of the genus, which can be further tested by a phylogenetic analysis. Even considering other batrachosauroidids from North America, comparable body size and combination of characters such as the unique shape of the Meckelian groove as well as the size and shape of the odontoid process of the atlas cannot be found in other batrachosauroidid salamanders. The detailed study of the articulated skeletal remains of the genus, such as the fossil remains of *Palaeoproteus klatti* from Geiseltal, Germany (middle Eocene)^23^ will be the key of resolving the phylogenetic relationships of the species within the genus.

In Europe, the fossil record of *Palaeoproteus* is limited to the late Paleocene to middle Eocene and some probable batrachosauroidids have been mentioned from the Cretaceous (Table 1). Though the palaeoherpetofauna of the late Eocene – Miocene of Europe has been rather extensively studied^25–28^, Batrachosauroididae have never been reported in younger localities. *Palaeoproteus miocenicus* sp. nov. appears in the fossil record of the genus after a gap of 30 million years, suggesting a hidden late Eocene–Miocene record of the family in Europe. Interestingly, the new species differs significantly in morphology from the two Paleogene species, suggesting a significant size enlargement within 30 million years. Most probably, *Palaeoproteus*, with its scarce and patchy stratigraphic record, represents a Lazarus taxon, with still undiscovered Cenozoic fossil record in Europe.

**Table 1:** Fossil record of batrachosauroidids in Europe.

locality	country	latitude	longitude	age (in Ma)	taxon	literature
Schernham	Austria	48.1500	15.5000	8.9–9.1	*Palaeoproteus miocenicus*	This study
Götzendorf	Austria	48.0167	16.5833	9.7–9.9	*Palaeoproteus miocenicus*	This study
Grytsiv	Ukraine	49.9750	27.1600	11.146–11.056	*Palaeoproteus miocenicus*	This study
Geiselthal, III Zone, Mittelkohle	Germany	51.30	11.90	43.5–47.8	*Palaeoproteus klatti*	^34^
Cernay	France	49.266	74.100	56–57.2	*Palaeoproteus gallicus*	^11^
Rilly	France	49.2	4.1	56–58	*Palaeoproteus gallicus*	^11^
Berru	France	49.2665	4.1355	56–58	*Palaeoproteus gallicus*	^11^
Hainin	Belgium	50.433	33.7500	61.7–62.5	*Palaeoproteus gallicus*	^35,36^
Champ-Garimond (Fons 0)	France	43.918	4.1774	72.1–83.6	? Batrachosauroididae indet.	^37,38^
Sunnydown Farm Quarry	England	50.6	2.0167	142.5–145.5	? Batrachosauroididae indet.	^39^

So far, batrachosauroidid salamanders have been found in Western and Central Europe (England, France and Germany) (Fig. 1). The present study extends the palaeogeographic distribution of both the genus *Palaeoproteus* and family eastwards, covering large areas from the easternmost parts of the Western Paratethys (Northern Alpine Foreland Basin) to Central (Pannonian Basin) and Eastern Paratethys. All three studied localities in Austria and Ukraine are characterized by wet climate. Mean annual precipitation values vary from 900 mm (Grytsiv 900 ± 252 mm, Schernham 1048 ± 258 mm, Table 2) to 1303 mm (Götzendorf 1303 ± 267 mm^29^). The depositional environment of Schernham and Götzendorf suggests the presence of wetland, floodplains and river, whereas in Grytsiv quiet sedimentation in karstic cavities has been suggested. Older localities with *Palaeoproteus* also suggest fully aquatic environments; e.g. lake for loc. Geiseltal^23^, river deposits for the localities Cernay, Berru, Hainin^11^. A fully aquatic lifestyle for *Palaeoproteus* was suggested by Herre^23^ on the bases of the morphology and anatomy of *P. klatti* (extraordinary large body length, slender body with short extremities, presence of external gills). The presence of external gills in *P. klatti* was suggested by Herre^23^ on the bases of similarities between the configuration of the hyobranchial apparatus (presence of several ceratobranchial) of *P. klatti* and recent proteids. Although no complete skeletons of *P. gallicus* and *P. miocenicus* are available, the same aquatic lifestyle for these species can be assumed as for *P. klatti*, because of similarities among homologous bones available for all three species.

**Table 2:** Palaeoprecipitation values for the Schernham and Grytsiv localities estimated using the bioclimatic analysis of the amphibian and reptilian assemblages according to Böhme *et al*.^33^.

Locality	Schernham	Grytsiv
Country	Austria	Ukraine
Age (in Ma)	8.9–9.1	11.056–11.146
Taxon		
*Palaeoproteus miocenicus* sp. nov.	1	1
*Andrias scheuchzeri*	1	
*Ukrainurus hypsognathus*		1**
*Mioproteus* sp.	1	
*Mioproteus caucasicus*		1*
*Chelotriton* sp.		
*Chelotriton paradoxus*	0.3918	0.3918*
*Triturus* sp.		0.3918
*Triturus* cf. *marmoratus*		0.513
*Salamandra* sp.		0.3918
*Latonia* sp.	0.3918	0.3918*
*Latonia* cf. *gigantea*		
*Bufo bufo*	0.3918	0.513
*Bufotes* cf. *viridis*		0
*Hyla* sp.	0.3918	
*Palaeobatrachus* sp.	1	1*
*Pelophylax* ex. gr. *ridibundus*	0.513	0.513
*Rana* sp.	0.3918	0.3918*
*Pelobates* sp.	0.0917	0.0917*
*Testudo* sp.	0	
Geoemydidae indet.	1	
*Chelydropsis* sp.	1	
*Mauremys* sp.	1	
Gekkonidae indet.		0*
Lacertidae indet.		0*
*Lacerta* sp.	0	
Scincidae indet.	0	0
*Pseudopus pannonicus*		0*
*Ophisaurus* sp.	0	0*
*Anguis* sp.	0.0917	0.0917
Amphisbaenidae indet.		0.0917
Scolecophidia indet.		0.0917
*Albeneryx volynicus*		0.0917
*Albeneryx* cf. *volynicus*	0.0917	
*Bransateryx* sp.	0.0917	0.0917
*Eryx* sp.	0.0917	
Average	0.45	0.33
MAP (in mm)	1048.71	900.00
Recent MAP (in mm)	949.80	492.00
Error (in mm)	258.16	252.73
City/town with the value of recent MAP	Ries am Innkre	Hrytsiv
error index	1.08	1.03
MAP/MAPrecent (%)	110.41	182.93
Literature	This study	This study

Faunal list based primarily on our examination of collections, plus relevant literature for Grytsiv^12,40^. The latter are indicated by one asterisk for Roček^40^ and double asterisk for Vasilyan *et al*.^12^.

The phylogenetic relationship of Batrachosauroididae relative to other salamander families is still poorly understood. The phylogenetic analysis based only on the vertebra morphology placed the family as a sister group to the Amphiumidae + Sirenidae clade^30,31^. Skutschas & Gubin^6^ (strict consensus of six most parsimonious trees) placed it as a sister clade to Proteidae + Sirenidae families. In Marjanović & Laurin^32^ batrachosauroidids are placed with an unresolved relationship with three further salamander clades (Scapherpetonidae, Cryptobranchoidea and Diadectosalamandroidei) within Urodela. For the better understanding of the phylogenetic relationship of the Batrachosauroididae to other salamander clades as well as among batrachosauroidid genera, a comprehensive study of *Palaeoproteus klatti* (represented by complete skeletons) from the Geiseltal locality, Germany, will be crucial.

The present study shows that, although in the last decades numerous studies have extensively documented the amphibian Cenozoic record, the fossil diversity of the amphibian fauna is far to be fully understood. The patchy stratigraphic occurrence of *Proteus miocenicus* as well as *Palaeoproteus* (as Lazarus taxa) may be explained by their narrow climatic and environmental spaces, which can be tested by comprehensive analysis of the entire faunistic assemblage, climatic and environmental conditions and taphonomic peculiarities of the fossiliferous horizons. Also potentially informative will be palaeobiogeographic analyses at an inter-continental scale (North America vs. Europe). Further studies and critical revisions of fossil material could provide new insights into uncovered fossil record of different vertebrate groups, extending their spatial and temporal distributions in the fossil record.

## Material and Methods

The studied fossil material is represented by isolated skeletal elements, which were collected by screen washing of sediments. The bones were photographed using a digital microscope Keyence (Fribourg, Switzerland). Some of the bones (Figs. 2–4) from the Schernham localities were CT scanned using the Bruker Skyscanner 2211 at the Univertsité de Fribourg, Switzerland with the following settings: 19 µm resolution, 206 mA, 89 kV, 0.5 mm Al filter. The tomographic reconstructions were performed using the Nricon software in Fribourg, Switzerland. The consequent visualisation and segmentation of the bone material were performed using the Amira 9.0 software in Porrentruy, Switzerland. The osteological nomenclature follows Gardner *et al*.^10^ and Vasilyan *et al*.^12^. The palaeoprecipitation estimates have been calculated using the bioclimatic method of Böhme *et al*.^33^. The methodology of the body size reconstructions/estimations can be found in the Supplementary Material.

The fossil material from the Austrian localities is stored in the collection of the Naturhistorisches Museum Wien, Vienna, Austria (NHMW), whereas material from the Ukrainian locality is at the National Museum of Natural History, Kyiv, Ukraine (NMNHU).

### Nomenclatural acts

This published work and the nomenclatural acts it contains have been registered in ZooBank, the proposed online registration system for the International Code of Zoological Nomenclature (ICZN). The ZooBank LSIDs (Life Science Identifiers) can be resolved and the associated information viewed through any standard web browser by appending the LSID to the prefix “http://zoobank.org/”. The LSIDs for this publication are urn:lsid:zoobank.org:pub:4EC994B3-70B1-449C-A02D-A20597384969.

## Supplementary information

Supplementary material.

## Data Availability

The fossil material from the Austrian localities is stored in the collection of the Naturhistorisches Museum Wien, Vienna, Austria (NHMW), whereas the Ukrainian locality at the National Museum of Natural History, Kyiv, Ukraine (NMNHU).
