# Supplementary material for: The last Palaeoproteus (Urodela: Batrachosauroididae) of Europe
Source: Sci Rep. 2020 Feb 17;10:2733. doi: 10.1038/s41598-020-59255-1 (PMC7026125; doi:10.1038/s41598-020-59255-1)
Supplement: Supplementary file 1 — Supplementary material. [file 41598_2020_59255_MOESM1_ESM.docx]

**Title: The last *Palaeoproteus* (Urodela: Batrachosauroididae) of Europe**

**Authors**

Davit Vasilyan^1,2,*^, Vadym Yanenko^3^

**Affiliations**

^1^JURASSICA Museum, Route de Fontenais 21, 2900 Porrentruy, Switzerland

^2^Department of Geosciences, University of Fribourg, Chemin du musée 6, 1700 Fribourg, Switzerland

^3^Department of Paleontology, National Museum of Natural History, National Academy of Sciences of Ukraine, 01030 Bogdan Khmelnitsky st. Kyiv, Ukraine

^*^corresponding author. Email: davit.vasilyan@jurassica.ch

**Supplementary Method M1.**

Estimation of the body length values

We have reconstructed the values of the total body length and snout-vent length of *P. miocenicus* and *P. gallicus* based on the length of the centra of the trunk vertebra. The publish data on *P. klatti*^1^ has been taken as a reference for the calculation of the ratios of the snout-vent length to the total body length. The ratio of the length of the vertebral centrum length to the body total length have been calculated on the complete skeletons of the *P. klatti* (Geiseltal, Germany, Eocene). For the estimation of the body lengths, we have taken the average values of the SVL/TL and VrtL/TL with calculation of their standard deviation values. The estimated values have been considered for the illustration (Figure 5) of the maximum body sizes of *Palaeoproteus* spp.

Supplementary table S1

Table S1. Morphometric measurements and meristic features of the *Paleoproteus* spp. The data on *P. klatti* is given according to Herre^1^. For *P. gallicus*, we have considered only all available trunk vertebrae (C.R. No. 6692) besides the first trunk vertebra (C.R. No. 6693)^2^. The reconstructed (estimated) values of the TL and SVL are indicated in blue and yellow colours accordingly. The highlighted values of TL and SVL are used for the illustration of the body sizes in Fig. 5. Abbreviation: TL, total length; SVL, snout-vent length; VrtL, vertebral centrum length.

| species | locality | coll nr. | TL  (in mm) | SVL  (in mm) | VrtL | SVL/TL ratio (%) | VrtL/TL ratio (%) |
| --- | --- | --- | --- | --- | --- | --- | --- |
| *P. klatti* | Geiseltal | 900 | 122 | 86 | - | 70.5 | - |
|  |  | 12 | 134 | 100 | - | 74.6 | - |
|  |  | 31 | 134 | 97 | - | 71.6 | - |
|  |  | 6 | 150 | 115 | - | 76.6 | - |
|  |  | 51 | 165 | 115 | - | 69.7 | - |
|  |  | 471 | 165 | 125 | 3.3 | 75.7 | 1.9 |
|  |  | 20 | 175 | 125 | 3.1 | 74.9 | 1.7 |
|  |  | 74 | 180 | 125 | - | 69.4 | - |
|  |  | 323 | 185 | 130 | 3.3 | 70.3 | 1.8 |
|  |  | 700 | 185 | 135 | - | 73 | - |
|  |  | 212 | **270** | **198**±7 | 5 | - | 2 |
|  |  | ranges | | | | 69.4-76.6 | 1.7-2 |
|  |  | mean±SD | | | | 73±2.67 | 1.85±0.01 |
| *P. gallicus* | Cernay | C.R. No. 6692 | **162**±1 | **118**±4 | 3 | - | - |
| *P. miocenicus* sp. nov. | Schernham | NHMW 2018/0290/0003 | 308±2 | 225±8 | 5.7 | - | - |
|  |  | NHMW 2018/0290/0004 | 389±3 | 284±10 | 7.2 | - | - |
|  |  | NHMW 2018/0290/0005 | 173±1 | 126±5 | 3.2 | - | - |
|  | Grytsiv | NMNHU-P 22-2708 | **389**±3 | **284**±10 | 7.2 | - | - |

References

1. Herre, W. Die Schwanzlurche der mitteleocänen (oberlutetischen) Braunkohle des Geiseltales und die Phylogenie der Urodelen unter Einschluß der fossien Formen. *Zoologica - Original-Abhandlungen aus dem Gesamtgebiete der Zoologie* **87,** 1–85 (1935).

2. Estes, R., Hecht, M. K. & Hoffstetter, R. Paleocene amphibians from Cernay, France. *American Museum Novitates* **2295,** 1–25 (1967).
